# KIDNEY-PAGER: analysis of circulating tumor DNA as a biomarker in renal cancer – an observational trial. Study protocol

**DOI:** 10.2340/1651-226X.2024.25581

**Published:** 2023-02-23

**Authors:** Laura Iisager, Johanne Ahrenfeldt, Anna Krarup Keller, Tommy Kjærgaard Nielsen, Niels Fristrup, Iben Lyskjær

**Affiliations:** a Department of Molecular Medicine, Aarhus University Hospital, Aarhus, Denmark; b Department of Urology, Aarhus University Hospital, Aarhus, Denmark; c Department of Urology, Aalborg University Hospital, Denmark; d Department of Oncology, Aarhus University Hospital, Aarhus, Denmark

**Keywords:** Renal cell carcinoma, translational research, biomarkers, circulating tumor DNA, risk stratification, minimal residual disease, liquid biopsy

## Abstract

**Background:**

Management of localized renal cell carcinoma (RCC) is challenged by inaccurate methods to assess the risk of recurrence and deferred detection of relapse and residual disease after radical or partial nephrectomy. Circulating tumor DNA (ctDNA) has been proposed as a potential biomarker in RCC.

**Purpose:**

Conduction of an observational study to evaluate the validity of ctDNA as a biomarker of the risk of recurrence and subclinical residual disease to improve postoperative surveillance.

**Material and methods:**

Urine and blood will be prospectively collected before and after surgery of the primary tumor from up to 500 patients until 5 years of follow-up. ctDNA analysis will be performed using shallow whole genome sequencing and cell-free methylated DNA immunoprecipitation sequencing. ctDNA levels in plasma and urine will be correlated to oncological outcomes. Residual blood and urine as well as tissue biopsies will be biobanked for future research.

**Interpretation:**

Results will pave the way for future ctDNA-guided clinical trials aiming to improve RCC management.

## Background

### Treatment of localized renal cancer

Localized renal cell carcinoma (RCC) is treated with partial or radical nephrectomy, or ablation therapy with curative intent. After surgery, patients with stage I–III RCC are offered a watchful waiting program depending on the given risk assessment. The postoperative follow-up scheme in Denmark relies on the Leibovich score which divides patients into ‘low’, ‘intermediate’, and ‘high’ risk groups. Based on this risk assessment, patients are offered computerized tomography (CT) scans at set intervals [1]. However, especially the ‘intermediate’ risk group constitutes a clinical challenge and could benefit from a refinement to identify which patients are most likely to relapse. In Denmark, roughly 25% of patients in the ‘intermediate’ risk group will relapse within 5 years, while the number is ~50% for the ‘high’ and ~10% for the ‘low’ risk group patients [2]. Thus, the current risk stratification is not optimal to identify patients with the highest risk of relapse after treatment of localized RCC.

Disease recurrence is closely linked to minimal residual disease (MRD) [3], thus detection of MRD early is critical for a successful treatment. However, no detection method for MRD is currently used in routine clinical practice [1], since small lesions and MRD might be undetectable with current imaging modalities which can only detect cancer nodules above a given size. Thus, there is a need for novel methods suitable for MRD detection.

### The potential of circulating tumor DNA in renal cancer management

Solid tumors, including RCC, release DNA fragments into the circulation. The tumor-derived part of the cell-free DNA (cfDNA), that circulates in biological fluids of cancer patients, is referred to as circulating tumor DNA (ctDNA). The half-life of cfDNA is short (<2 h) [4], enabling real-time characterization of tumor burden, with detection of ctDNA postoperatively indicating residual disease or occult dissemination of cancer cells prior to surgery.

Earlier studies have proposed cfDNA/ctDNA as a prognostic biomarker and a monitoring tool in RCC (reviewed in [5]). Until recently, ctDNA assays were challenged by the trace amounts of ctDNA shed by RCC [5, 6]. However, with the new more sensitive method cell-free methylated DNA immunoprecipitation-sequencing (cfMeDIP-seq) it is now feasible to study ctDNA in patients with RCC [7].

### Rationale

The hypothesis of the KIDNEY-PAGER study is that ctDNA detected before and/or after intended curative treatment for localized RCC can be utilized as a biomarker predicting higher risk of recurrence, indicating subclinical MRD, and as a sensitive tool for monitoring recurrence during postoperative surveillance.

### Aims and objectives

The overall aim of this protocol is to conduct an observational study to confirm that ctDNA detected in plasma and/or urine after treatment for RCC with curative intent can be applied in clinical practice as a biomarker of subclinical MRD and risk of recurrence. Thus, this study aims to pave the way for future ctDNA-guided clinical trials with the aim of improving postoperative surveillance and treatment strategies for patients with RCC.

#### Primary objective

1. To confirm that patients with residual disease and a high risk of recurrence can be identified with ctDNA profiling performed immediately after nephrectomy.

#### Secondary objectives

1. To apply ctDNA pre- and post-operatively as a risk stratification tool.
2. To validate the potential of a ctDNA-guided follow-up scheme compared to the current follow-up scheme with frequent CT scans after surgery.
3. To investigate a possible lead-time between *molecular* recurrence (detected using serial plasma and urine ctDNA analyses) and *clinical* recurrence (detected using radiological examinations).
4. To find and validate prognostic and predictive blood-based biomarkers for immunotherapy and/or targeted therapies by correlating ctDNA levels with oncological outcome parameters.
5. To confirm that changes in ctDNA levels reflect the therapeutic effect of the given therapy.
6. To delineate markers of tumor aggressiveness and compare them to ctDNA measurements.

## Material and methods

### Study design

**Figure 1:**
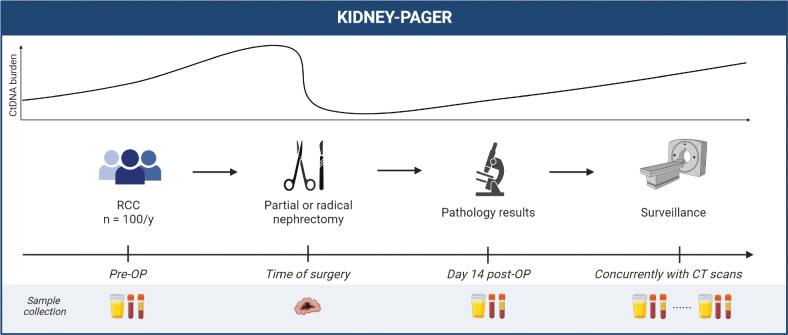
Study design. Blood (plasma and buffy coat), urine, and tumor tissue will be collected as indicated during patient management. CT: computed tomography; pre-OP: preoperatively; post-OP: postoperatively; RCC: renal cell carcinoma. Figure is created with BioRender.com.

This study is based on a prospective collection of blood and urine samples for ctDNA analysis before and after surgery of localized RCC and during postoperative surveillance (Figure 1). Included patients are in the project followed until the 5-year scheduled follow-up CT scan.

1. Urine and blood samples for ctDNA analysis were taken preoperatively and approximately 2 weeks after surgery.
2. Sampling of tissue from the resected tumor specimen as well as adjacent normal kidney.
3. Longitudinal blood and urine sampling is contemporary with follow-up abdominal and thoracic CT scans, and with oncological treatment if metastatic disease is diagnosed. If relapse occurs, tissue from metastatic lesions will be obtained if possible.

### Study population and eligibility criteria

During the inclusion period, we will prospectively include patients treated for RCC with curative intent at Aarhus University Hospital (AUH), Denmark. In total, a maximum of 500 patients operated for RCC will be included with the expectation of up to 100 patients being included per year for 5 years. The following eligibility criteria will be applied:

#### Inclusion criteria

- Patients diagnosed with a locoregional renal cancer are eligible for surgical excision with curative intent and with sufficient performance status for surgery.
- Patients with metastatic disease, but no evidence of disease after surgery and local treatment of metastases.
- Patients aged 18 years or older.
- Patients must able to understand and sign written informed consent.

#### Exclusion criteria

- Patients with local disease who are not being offered surgical treatment, including ablation therapy.
- Patients who are unlikely to comply with the protocol (e.g. uncooperative attitude), inability to return for subsequent visits, and/or otherwise considered to be unlikely to complete the study by the investigator.

### Recruitment and patient consent

Patients are approached in person at the treating Department of Urology, AUH. Here, patients are given written information regarding the project from which they can give informed consent. Furthermore, as the project involves genomic sequencing, which may disclose genetic variants predisposing to specific diseases, participants are given the opportunity to refuse information upon findings of special germline variants. The informed consent may be withdrawn at any time without having any impact on current or future treatment. This study will not present any personal data in any way and will not require consent for publication by any participant.

### Status

Inclusion began in June 2023 and is expected to end in 2028. The primary endpoint will be reached and ready for publication in 2031. The planned 5 years of follow-up will be complete and available for all patients by 2033. The study has been registered in the Clinical Trials database: NCT06145139.

### Sample collection

All patients will have one blood sample drawn prior to and post-surgery. The latter will take place when the patients visit the Urological Department to get the results from the histopathological examination of the resected tumor, usually on day 14. Additional blood samples will be drawn longitudinally during the postoperative follow-up period simultaneously with the planned CT scans (the number and timing of scans depends on the assigned risk group). Additionally, all patients will be asked to give a urine sample on the same dates as blood samples are drawn.

Whenever possible, fresh tumor and adjacent normal parenchyma biopsies will be collected from the surgical specimen.

### Experimental plans and statistically analysis

ctDNA will be detected and measured before and after surgery using shallow whole genome sequencing (sWGS) and cfMeDIP-sequencing [8]. ctDNA levels will be estimated from sWGS data by the assessment of copy number profiles. Enrichment data from cfMeDIP-sequencing will be transformed into methylation levels and normalized to copy number variants in the sWGS data. Differentially methylated regions will be called whereafter a classifier will be built using machine learning methods. ctDNA levels will be estimated from methylation data by assignment of a methylation score to each patient sample based on the established classifier. ctDNA detection will be correlated with time to relapse, progression-free survival, time to subsequent systemic therapy, and overall survival, using statistical methods (e.g. Cox regression). Moreover, ctDNA levels will be measured longitudinally during surveillance to detect relapses. ctDNA detection will be correlated with the clinical detection of relapse using CT scans, and if applicable, lead time between molecular recurrence and clinical recurrence will be calculated.

### Biobanking and collection of clinical information

The collection of samples is performed in collaboration with the Bio- and Genome Bank Denmark (RBGB). Residual blood, urine, and tissue biopsies as well as clinical and sequencing data will be transferred to the Renal Cancer Research Biobank at AUH for future research. The material in the research biobank will be saved and stored in a pseudonymised form at the Department of Molecular Medicine, AUH in Denmark. The samples will be marked with a unique patient number, and only the project responsible will be able to connect this number with the patient social security number. A central part of the project involves comparison and correlation of ctDNA assessments (gathered from the blood and urine samples) with the clinical information on treatment and outcome. Therefore, clinical information regarding the treatment and outcome of the treatment will be collected from hospital records and health registries; specifically, information about surgical intervention, pathology reports, biomarkers of inflammation, biomarkers of kidney function, oncological intervention, and radiological evaluations aiming at detecting disease recurrence or assessing changes in tumor burden during surveillance and treatment. Moreover, results from blood analyses will be noted.

### Ethical considerations

The ethical concerns and risks related to this study are limited. Blood sampling is associated with minimal risk and discomfort to the patient. Urine sampling is safe for the patient. Tissue samples are collected from the specimens that are resected as part of the standard treatment of the patients or to confirm disease recurrence. Hence, these biopsies introduce no additional risk to patients.

### Publication policy

Positive, negative, and non-definable findings from KIDNEY-PAGER will be published in peer-reviewed scientific journals.

## Discussion

Identifying patients with a greater risk of relapse after surgery for localized RCC could improve oncological outcomes. Recurrence usually occurs within 3 years of surgery [9], and currently the Leibovich score is used in Denmark for risk assessment. However, the current risk assessment is not sufficient to identify patients with the highest risk of relapse after surgery for localized RCC as patients across all three risk groups develop disease recurrencies [2, 10]. To improve monitoring of recurrencies, we suggest analyzing ctDNA in the management of RCC. A study from 2020 revealed the ability of detecting ctDNA across all RCC stages upon the use of the sensitive enrichment-based cfMeDIP-seq technique, despite the low amount of ctDNA shed from RCC [7].

The expected outcome of KIDNEY-PAGER is an assessment of the validity of ctDNA-based analysis to provide a more tailored postoperative follow-up scheme for RCC patients who had undergone surgery for localized RCC with curative intent. If positive for ctDNA, patients may benefit from a closer follow-up scheme regardless of the Leibovich score or would even need an immediate intervention, such as adjuvant therapy. On the contrary, surveillance, including serial ctDNA analyses instead of regular scans, could potentially be sufficient for ctDNA-negative patients, and this could consequently prevent unnecessary use of imaging resources as well as save the ctDNA-negative patients from needless worries. Yet, caution is essential to mitigate the risk of false negatives arising from technical challenges like minimal shedding from tumors. Therefore, it is advisable to consider ctDNA primarily as a supplementary tool to complement radiological evaluations in the initial stages.

Naturally, given the recent approval of pembrolizumab by the European Medicines Agency (EMA) as an adjuvant treatment for adult patients with RCC at intermediate-high or high risk of recurrence after nephrectomy, or following nephrectomy and the complete resection of metastatic lesions, ctDNA could potentially be used to stratify patients for adjuvant treatment. Yet the approval of pembrolizumab is still pending in Denmark. ctDNA analysis can further enable the improvement of factors associated with suboptimal surgery as well as the identification of patients having MRD who may benefit from adjuvant therapy but are left untreated today (stages I, II, III). The clinical benefit of treating low stage ctDNA-positive patients with adjuvant therapy needs to be further studied in randomized clinical trials.

In addition to the more accurate assessment of risk for relapse after surgery, the clinical implication of ctDNA monitoring could be the early detection of clinical relapses and residual disease while the lesions are small and potentially responsive to local or systemic therapies. This will potentially improve the disease-specific survival of patients with RCC in the future.

## Data Availability

The datasets used and/or analyzed during the current study will be available from the corresponding author on reasonable request.

## References

[1] Leibovich BC, Blute ML, Cheville JC, et al. Prediction of progression after radical nephrectomy for patients with clear cell renal cell carcinoma: a stratification tool for prospective clinical trials. Cancer. 2003;97:1663–71. 10.1002/cncr.11234 12655523

[2] Azawi NH, Tesfalem H, Mosholt KSS, et al. Recurrence rates and survival in a Danish cohort with renal cell carcinoma. Dan Med J [Internet]. 2016;63. Available from: https://www.ncbi.nlm.nih.gov/pubmed/27034180 [cited: 2023-11-08] 27034180

[3] Larribère L, Martens UM. Advantages and challenges of using ctDNA NGS to assess the presence of minimal residual disease (MRD) in solid tumors. Cancers. 2021;13:5698. 10.3390/cancers13225698 34830853 PMC8616165

[4] Corcoran RB, Chabner BA. Application of cell-free DNA analysis to cancer treatment. N Engl J Med. 2018;379:1754–65. 10.1056/NEJMra1706174 30380390

[5] Geertsen L, Koldby KM, Thomassen M, Kruse T, Lund L. Circulating tumor DNA in patients with renal cell carcinoma. A systematic review of the literature. Eur Urol Open Sci. 2022;37:27–35. 10.1016/j.euros.2021.12.006 35106503 PMC8784339

[6] Smith CG, Moser T, Mouliere F, et al. Comprehensive characterization of cell-free tumor DNA in plasma and urine of patients with renal tumors. Genome Med. 2020;12:23. 10.1186/s13073-020-00723-8 32111235 PMC7048087

[7] Nuzzo PV, Berchuck JE, Korthauer K, et al. Detection of renal cell carcinoma using plasma and urine cell-free DNA methylomes. Nat Med. 2020;26:1041–3. 10.1038/s41591-020-0933-1 32572266 PMC8288043

[8] Shen SY, Burgener JM, Bratman SV, De Carvalho DD. Preparation of cfMeDIP-seq libraries for methylome profiling of plasma cell-free DNA. Nat Protoc. 2019;14:2749–80. 10.1038/s41596-019-0202-2 31471598

[9] Diaz de Leon A, Pirasteh A, Costa DN, et al. Current challenges in diagnosis and assessment of the response of locally advanced and metastatic renal cell carcinoma. Radiographics. 2019;39:998–1016. 10.1148/rg.2019180178 31199711 PMC6677287

[10] Lyskjær I, Iisager L, Axelsen CT, Nielsen TK, Dyrskjøt L, Fristrup N. Management of renal cell carcinoma: promising biomarkers and the challenges to reach the clinic. Clin Cancer Res. 2023. 10.1158/1078-0432.CCR-23-1892 PMC10870122 37874628

